# Extraintestinal Pathogenic *Escherichia coli*: Virulence Factors and Antibiotic Resistance

**DOI:** 10.3390/pathogens10111355

**Published:** 2021-10-20

**Authors:** Valerio M. Sora, Gabriele Meroni, Piera A. Martino, Alessio Soggiu, Luigi Bonizzi, Alfonso Zecconi

**Affiliations:** Surgical and Dental Sciences-One Health Unit, Department of Biomedical, School of Medicine, University of Milan, Via Pascal 36, 20133 Milan, Italy; gabriele.meroni@unimi.it (G.M.); piera.martino@unimi.it (P.A.M.); alessio.soggiu@unimi.it (A.S.); luigi.bonizzi@unimi.it (L.B.); alfonso.zecconi@unimi.it (A.Z.)

**Keywords:** *Escherichia coli*, ExPEC, virulence factors, antibiotic resistance

## Abstract

The One Health approach emphasizes the importance of antimicrobial resistance (AMR) as a major concern both in public health and in food animal production systems. As a general classification, *E. coli* can be distinguished based on the ability to cause infection of the gastrointestinal system (IPEC) or outside of it (ExPEC). Among the different pathogens, *E. coli* are becoming of great importance, and it has been suggested that ExPEC may harbor resistance genes that may be transferred to pathogenic or opportunistic bacteria. ExPEC strains are versatile bacteria that can cause urinary tract, bloodstream, prostate, and other infections at non-intestinal sites. In this context of rapidly increasing multidrug-resistance worldwide and a diminishingly effective antimicrobial arsenal to tackle resistant strains. ExPEC infections are now a serious public health threat worldwide. However, the clinical and economic impact of these infections and their optimal management are challenging, and consequently, there is an increasing awareness of the importance of ExPECs amongst healthcare professionals and the general public alike. This review aims to describe pathotype characteristics of ExPEC to increase our knowledge of these bacteria and, consequently, to increase our chances to control them and reduce the risk for AMR, following a One Health approach.

## 1. Introduction

Antimicrobial resistance (AMR) is a natural phenomenon linked to the creation and spread of resistance factors enhanced using antibiotics. The One Health approach emphasizes the importance of AMR’s presence as a major concern both in public health and in food animal production systems [1,2]. Among the pathogens, *E. coli* is becoming of great importance, and it has been suggested that both Extraintestinal Pathogenic *E. coli*, (ExPEC) and commensal *E. coli* (CoEC) strains may harbor resistance genes that may be transferred to pathogenic or opportunistic bacteria [3,4]. Specifically, ExPEC strains are also gaining new and troubling antibiotic resistance genes [5]. Indeed, in this context of rapidly increasing multidrug-resistance worldwide and a diminishingly effective antimicrobial arsenal to tackle resistant strains, ExPEC infections are now a serious public health threat worldwide. However, the clinical and economic impact of these infections and their optimal management are challenging and, consequently, there is an increasing awareness of the importance of ExPECs amongst healthcare professionals and the general public alike [6,7,8]. This review aims to supply an organized description of ExPEC pathotypes characteristics to increase the knowledge of these bacteria and, consequently, increase our chances of controlling them and reducing the risk for AMR, following a One Health approach.

## 2. *Escherichia coli*

*Escherichia coli* is a Gram-negative bacterium, facultative anaerobe, not sporogenous, belonging to the *Enterobacterales* order. It has a rod shape of roughly 0.4 μm in diameter and 2–3 μm in length. It can be provided or not with a capsule and is generally mobile due to the peritric flagella. It grows in a wide range of temperatures (15–45 °C) and can survive in the environment for long periods [9].

*E. coli* is the main facultative anaerobe in the intestinal flora of humans and warm-blooded animals and is generally non-pathogenic. Most of the *E. coli* strains isolated from 1895 to today are part of the commensal microbial population of the intestine; it is typically present in feces, in the amount of 10⁷–10⁹ CFU/g and is usually the predominant microorganism in coprocultures of all mammals. Nevertheless, specific strains have developed pathogenetic mechanisms capable of causing several pathologies, even severe ones in humans and animals [10,11].

Strains of *E. coli* are serologically classified according to the antigenic differences of the 173 O (somatic) and 56 H (flagellar) antigens, based on the typing scheme introduced in 1947 by Kauffmann and still recognized and used internationally [12]. The set of bacterial strains characterized by the same O antigen constitutes a "serogroup", while a specific combination of O antigen and H antigen defines the "serotype" of an isolate. Additional antigens analyzed in the Kauffmann classification are the K (capsular) and F (fimbrial) antigens [9].

The clustering of an *E. coli* strain to a specific serogroup or serotype can be associated with the presence of certain pathogenetic factors and the ability to cause specific symptoms. However, the serotype alone cannot predict either the genotypic characteristics or the pathogenicity since the horizontal gene transfer allows strains with identical phenotypic characteristics (i.e., the serotype) to have several genotypic differences (i.e., different pathogenic factors). More recently, in addition to Kaufmann’s classification scheme, *E. coli* have been classified into pathotypes based on their pathogenetic profile, which considers the virulence factors and the diseases caused mainly in humans [13]. Indeed, pathogenic *E. coli* can be distinguished from their non-pathogenic counterparts by the presence of virulence genes, which code for adherence, colonization, invasion, cell surface molecules, secretion, transport, and siderophore formation. These virulence genes are generally organized as large blocks in chromosomes, plasmids, or phages and are often transmissible between *E. coli* strains.

## 3. *Escherichia coli* Pathotypes 

**Figure 1:**
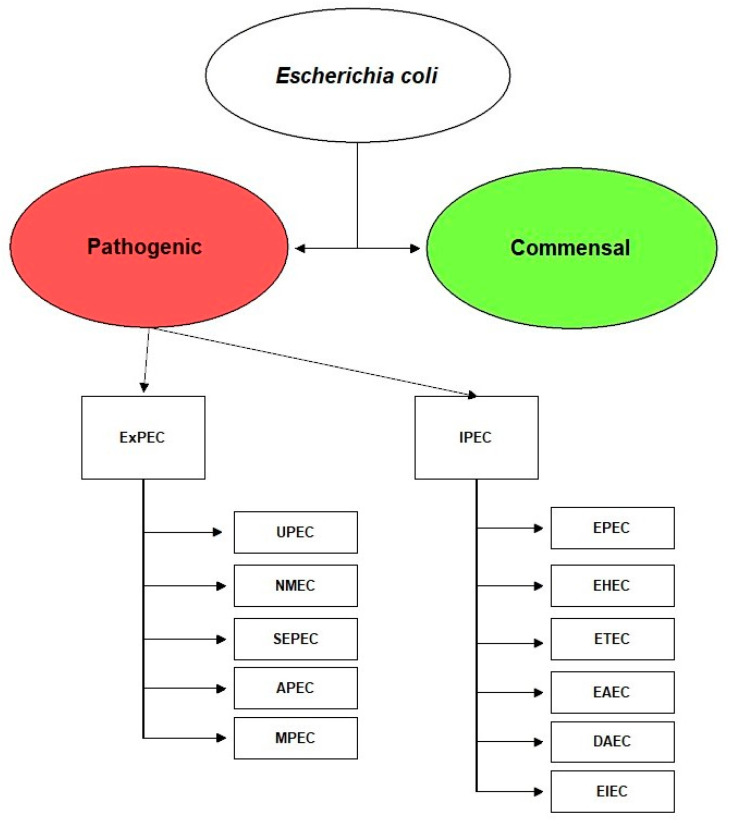
Classification of extraintestinal and intestinal pathogenic *E. coli* variants. Abbreviations: intestinal pathogenic *E. coli* (IPEC), enteropathogenic *E. coli* (EPEC), enterohemorrhagic *E. coli* (EHEC), enterotoxigenic *E. coli* (ETEC), enteroaggregative *E. coli* (EAEC), diffusely adherent *E. coli* (DAEC), enteroinvasive *E. coli* (EIEC), extraintestinal pathogenic *E. coli* (ExPEC), uropathogenic *E. coli* (UPEC), neonatal meningitis *E. coli* (NMEC), sepsis-associated *E. coli* (SEPEC), avian pathogenic *E. coli* (APEC), and mammary pathogenic *E. coli* (MPEC).

Based on this latter classification, *E. coli* can be distinguished into two main groups based on the ability to cause infection of the gastrointestinal system (Intestinal Pathogenic *Escherichia coli*, IPEC) or outside this (Extraintestinal Pathogenic *Escherichia coli*, ExPEC) (Figure 1). Among IPEC, *E. coli* strains are categorized into the following pathotypes: (1) enteropathogenic *E. coli* (EPEC), which causes diarrhea in children and animals; (2) enterohemorrhagic *E. coli* (EHEC), responsible for hemorrhagic colitis and hemolytic-uremic syndrome; (3) enterotoxigenic *E. coli* (ETEC), the main cause of traveler’s diarrhea and porcine and bovine diarrhea; (4) enteroaggregative *E. coli* (EAEC), that can cause persistent diarrhea in humans, and (5) diffusely adherent *E. coli* (DAEC), a subclass of enteroaggregative *E. coli* which causes diarrhea in children; (6) enteroinvasive *E. coli* (EIEC), which causes watery diarrhea and dysentery. EIEC and EAEC strains were found only in humans and not in animals [14]. 

The ExPEC group incorporates the following variants: uropathogenic *E. coli* (UPEC), neonatal meningitis *E. coli* (NMEC), those isolates responsible for septicemia (SEPEC), avian pathogenic *E. coli* (APEC) and mammary pathogenic *E. coli* (MPEC), [15]. These pathotypes are also isolated from animal diseases such as fatal pneumonia in pigs, mastitis in cows and pigs, pyometra, and urinary tract infections in dogs [16,17,18].

## 4. Extraintestinal Pathogenic *Escherichia coli*, ExPEC 

ExPEC strains are versatile bacteria that can cause urinary tract, bloodstream, prostate, and other infections at non-intestinal sites. ExPEC strains are responsible for many human infections, both in healthcare-associated and community settings [19,20]. They typically occupy a niche in the intestinal microbiota of humans (and other animals), and from this reservoir, they emerge to cause extraintestinal infections [5]. Aside from these bacteria-specific traits, additional factors are required for illness (i.e., host-specific factors) are also required for disease, and for this reason, ExPEC are considered a necessary but not sufficient cause for extraintestinal *E. coli* infections [21], and a limited set of ExPEC lineages are responsible for most infections [22]. 

ExPECs are most frequently implicated as urinary tract pathogens and they are the infective agent in up to 90% of both simple community-acquired urinary tract infections (UTIs) and pyelonephritis cases. Other urinary tract infections, including prostatitis and catheter-associated UTIs, are also frequently caused by ExPECs [19,23,24].

ExPECs are also implicated in infections originating from abdominal and pelvic sources, including, but not limited to, biliary infections, infective peritonitis, and pelvic inflammatory disease [25,26]. Hematogenous invasion of ExPEC from the initial infective focus results in sepsis syndrome, which may result in death in the absence of timely management [8].

## 5. ExPEC Virulence Factors

ExPEC virulence factors can be divided into five categories: adhesins, invasins, iron uptake factors, protectines, and toxins. The list of all the virulence factors and their presence within the different ExPEC pathotypes are reported in Table 1.

### 5.1. Adhesins

Many surface structures play a significant role in the process of specific adhesion. There are three main types of adhesins: fimbriae, afimbrial adhesins (Afa), and outer membrane proteins (OMPs) [28]. This family of adhesive and virulence-associated surface structures in Gram-negative bacteria is assembled and secreted by the chaperon-usher pathway, which involves the binding of nascent pilus subunits by a dedicated chaperone in the bacterial periplasm and the subsequent polymerization of subunits into the pilus fiber at the outer membrane by an integral outer membrane channel protein termed the usher [29]. The expression of surface adhesins increases the virulence of pathogenic *E. coli* by facilitating close contact of the bacteria with the host cell wall. Different bacterial adhesins are adapted to colonize specific niches. S-fimbrial adhesins (*sfa*), F1C (“pseudotype I“) fimbriae (*foc*), coding P-like pili, papC, and Iha (*iha*) are the most frequently detected adhesins among isolates from UTI patients [30]. For UPEC strains, P- and S- fimbriae receptors are located on the surface of epithelial cells lining the host urinary tract [31]. S-fimbrial adhesins are virulence factors present in strains responsible for meningitis and sepsis. These fimbriae can bind extracellular matrix components and sialoglycoproteins on brain capillary endothelial cells. NMEC is characterized by K1 capsular antigens (kpsM, neuA) or the *ibeA* invasion gene. The relationship between the type of infection and the presence of characteristic virulence factors has been demonstrated, i.e., IbeA protein has surface receptors on the brain capillary endothelial cells and allows pathogens to invade the nervous system [32].

Fimbriae with an affinity for structures containing mannose residues were classified as type 1, and mannose resistant fimbriae as type 2 (e.g., P, S, Dr fimbriae). *FimB* and *fimE* genes are responsible for controlling the expression of type 1 fimbriae. Three other genes, i.e., *fimF*, *fimG,* and *fimH* are involved in the adhesive property and longitudinal regulation. FimH adhesin, present on top of a fibrillum attached at the end of a pili rod composed of FimA proteins, binds to uroplakin 1A receptor (UP1a) of bladder epithelial cells, allowing invasion and formation of biofilm-like intracellular structures [33,34]. Mannose-resistant fimbriae include hemolytic F-type fimbriae, encoded by 11 genes in the *pap* genes cluster located on the chromosome. PapG adhesin occurs in 3 molecular variants: PapGI, PapGII, PapGIII, with PapGIII associated with bladder inflammation in women and children, and PapGII related to human bacteremia. The P fimbriae are also common virulence factors in renal transplant patients and patients with acute renal impairment [35,36]. According to Shetty et al. 2014, the high prevalence of adhesin-encoding genes from *E. coli* isolates in patients diagnosed with UTI confirms that these structures are necessary to cause an infection [27].

A significant group of superficial virulence factors found mainly in UPEC strains and associated with the occurrence of pyelonephritis and recurrent cystitis is the Afa/Dr family of adhesins, which contains both the fimbrial adhesins with Dr fimbriae and Afa [37]. *draA*, *draB*, *draC*, *draD,* and *draE* (*afaA*, *afaB*, *afaC*, *afaD, afaE*) genes encode a set of proteins responsible for the regulation of expression, activity as periplasmic chaperone, outer membrane usher, fimbrial tip subunit of unknown function, and adhesion/fimbrial subunit respectively. The receptor common for the whole family is the decay-accelerating factor (DAF), expressed on the surface of erythrocytes and cells of other tissues (e.g., the epithelium of the urinary tract) [38].

UpaG, a member of the auto-transporter family of adhesins, shows an affinity for fibronectin and laminin, allowing UPEC adherence to the bladder epithelium. Additionally, UpaG creates biofilm on plastics, which facilitates colonization of urological catheters [39]. Urinary tract infections can often cause bacteremia, especially in hospitalized patients, when biofilm is produced due to catheter contamination (36,37). Moreover, the translocation of bacterial cells from the growth phase in plankton to the growth phase in the biofilm is associated with a change in the expression of many genes (e.g., *flu* and *tibA*) encoding virulence factors and regulatory proteins. The biofilm-forming ability of *E. coli* is affected by the expression of gene encoding antigenic Agn43, which is involved in the aggregation of *E. coli* cells [40].

#### Biofilm

Biofilms are dynamic assemblages of microorganisms that adhere to biotic or abiotic surfaces and are characterized by highly specialized interactions between microorganisms. The bacteria are embedded in a matrix of self-produced extracellular polymeric substances (EPS) which allows one to alter phenotypes (e.g., growth rate, gene transcription, and antibiotic resistance) [41,42]. Schematically, a model for the formation of a differentiated and mature bacterial biofilm requires five developmental stages: (i) a reversible attachment of planktonic bacteria to the solid surface; (ii) transition from reversible to irreversible attachment due to production of EPS and/or specific adhesins located on pili and fimbriae, which interact with the surface (e.g., flagella, autotransporter proteins, fimbriae, curli, EPS, and F-type conjugative pilus) [43,44,45]; (iii) early development of biofilm architecture. (iv) late development of microcolonies into a mature biofilm; (v) dispersion and detachment of cells from the biofilm into the environment. All these processes need to be highly regulated, for this reason, bacteria have evolved different cell-to-cell signaling molecules that are the main constituent of signaling pathways responsible for population density-dependent gene expression and biofilm formation. In Gram-negative bacteria, the molecules devoted to the cell-to-cell signaling are acylated homoserine lactones (AHLs, like *N*-acyl-homoserine-lactone in *Vibrio fischeri*) expressed by *luxI* gene (firstly described in *V. fischeri*) that bind and activate LuxR protein and regulates the transcription of several genes involved in biofilm formation, expression of specific adhesins and metabolism [46]. *E. coli* (as well as *Salmonella*) does not contain *luxI* gene and is unable to synthesize AHLs; however, it expresses SdiA protein, similar to the LuxR-type transcriptional activator in AHL-dependent quorum-sensing, which makes *E. coli* biofilm regulation and synthesis susceptible to signaling molecules produced by other species [47]. Numerous *E. coli* strains can express the autoinducer-2 (AI-2) which is widespread in both Gram-negative and Gram-positive bacteria and regulates multiple functions such as the production of flagella and motility, but its expression is dependent on *luxS* gene product. The *luxS*/AI-2 system is one of the most widespread quorum-sensing systems (being described for the first time in *Vibrio harveyi*), LuxS is involved in the metabolism of S-adenosylmethionine (SAM) by converting in homocysteine and 4,5-dihydroxy-2,3-pentanedione (DPD), the cyclization of DPD generates several furanones including the precursor of AI-2 [46]. Different authors have demonstrated that AI-2 production is growth-phase dependent [48]. Currently, there are two types of AI-2 receptors, LuxP and LsrB, which were initially described in *V. harveyi* and *Salmonella typhimurium*, respectively [49]. *lsrB* gene is contained in the lsr operon, which consists of eight genes (lsrKRACDBFG) devoted to an ATP binding cassette (ABC) transporter synthesis. LuxP is homologous to the ribose binding protein of *E. coli* and *S. typhimurium* and acts as the primary sensor for AI-2 [50]. In ExPEC strains, the *tolC* gene (which belongs to the outer membrane efflux protein (OEP) family devoted to maintaining the structure and function of the outer membrane) is essential for biofilm formation in response to altered osmolarity also via depleted curli production [51].

### 5.2. Invasins

The adhesion of *E. coli* to membrane receptors of host cells triggers a cascade of host-cell signal transduction pathways that result in host-cell actin cytoskeleton rearrangements required for bacterial invasion of Human Brain Microvascular Endothelial Cells (HBMEC) [52]. Regulation of the rearrangement of the host actin cytoskeleton by *E. coli* K1 is a complex multifactorial process involving host actin-binding proteins and signaling molecules, as well as specific microbial determinants [52]. However, the underlying host-microbial interactions in the bacterial invasion of HBMEC remain not entirely understood [53].

The Rho family of small GTPases such as RhoA, Rac1, and CDC42 have been shown to regulate host cytoskeleton organization, acting as molecular switches, and various pathogens (e.g., *C. trachomatis, N. gonorrhoeae*) have been shown to exploit the Rho GTPases to aid their entry into the host cells [54,55].

Other invasins such as IbeA, belonging to the Pfam12831 family of FAD-dependent oxidoreductases and OmpA may contribute to HBMEC invasion via Rac1 activation [53]. The *ibeA* gene is located on the 20.3 kb island *GimA*, inserted between *yjiD* and *yjiE,* and proposed to contain four operons, some of which are potentially involved in energy metabolism [56]. This gene is not ubiquitous among *E. coli*; it is absent in non-pathogenic strains, [57,58] and encodes a protein that plays an essential role in the invasion of ExPEC in Brain Microvascular Endothelial Cells (BMEC) and the invasion of intestinal epithelial cells. This gene might be relevant for bacterial survival [59]. Indeed, the inactivation of *ibeA* in the NMEC and APEC strains has resulted in the defective invasion of BMEC both *in vitro* and *in vivo* [59,60,61,62]. It has been demonstrated that IbeA interacts with its receptor, an albumin-like protein, on the surface of both human (45 kDa) and bovine (55 kDa) BMEC. IbeA-binding protein was purified from intestinal epithelial Caco-2 cells [63,64,65,66]. IbeA could therefore be involved in the receptor ligand-mediated invasion of BMEC [58].

Considering what has been described before, it can be said that *ibeA* is involved in the pathogenesis of several ExPEC pathotypes, including NMEC and APEC, and contributes to the invasion of ExPEC into host cells [60,62]. Furthermore, the IbeA receptor PSF was described for both human and bovine BMEC [63,64,65,66].

The results obtained by Wang et al. 2011 indicated that the loss of *ibeA* resulted in reduced tissue-recovered bacteria than those of the wild-type strain [58]. Similarly, Germon et al. 2005 observed that the inactivation of *ibeA* caused the reduction of the presence of bacteria in cells and blood [60]. This phenomenon might be due to the downregulation of other virulence factors, such as type 1 fimbriae, autotransporter adhesin [67,68], and the invasion-associated gene *ibeB* in the mutant strain, concluding that all these genes participate in the adhesion and invasion of APEC to host cells [58]. The inactivation of *ibeA* decreases the expression level of virulence factors involved in invasion and adhesion, causing loss of colonization and proliferation capacities of APEC [58]. *IbeA*-negative strains showed impaired intramacrophage survival. This mechanism has not been elucidated anymore, but it is supposed that this gene could confer tolerance to reactive oxygen species (ROS) induced stress [69]. Finally, the presence of *ibeA* in biofilm-forming strains has been observed, but no studies have shown that this gene is involved in biofilm formation [58].

### 5.3. Iron Uptake Factors

Siderophores are secondary metabolites that capture iron to enhance bacterial growth and development [70]. Iron deficiency weakens bacterial adaptive abilities by generating disturbances in cellular capsules structure and synthesis [71]. In ExPEC, access to iron in the blood serum is of paramount importance. Since *E. coli* can cause sepsis and infections of various organs with very low iron availability, this pathotype has developed many strategies to obtain iron from infected sites [72,73].

Siderophores can be divided into five main classes: catecholates, phenolates, hydroxamic acids, α-hydroxycarboxylates, and a mixed type containing different siderophores. ExPECs are equipped with siderophores that increase their virulence: enterobactin and salmochelin (catecholate siderophores), aerobactin (a mixed-type siderophore), and yersiniabactin (phenolate siderophore).

#### 5.3.1. Enterobactin

Enterobactin is produced by nearly all *E. coli* strains and produced by many other pathogenic enterobacteria, including *Salmonella* spp. and *Klebsiella spp.* [74,75]. The genes responsible for enterobactin biosynthesis and transport are grouped in a single contiguous gene cluster *entEBG(AC)* [74]. Enterobactin biosynthesis is a two-step process involving six genes responsible for the synthesis of six corresponding proteins [76]. First, 2,3-dihydroxybenzoate (2,3-DHB) is synthesized from chorismate by EntC (isochorismate synthetase), EntB (isochorismatase), and EntA (dihydroxybenzoate deshydrogenase) [76,77,78,79]. Then, serine and 2,3-DHB are processed by an NRPS (nonribosomal peptide synthetase) enzymatic complex composed of EntB, EntE, and EntF, to form the final enterobactin molecule, corresponding to a cyclized DHBS (2,3-dihydroxybenzoylserine) triester [80,81,82,83,84]. This step also requires the phosphopantetheinyltransferase EntD to activate the thiolation domains of the NRPS systems and allow 2,3-DHB to bind to the assembly complex [81,85].

Enterobactin is found in both pathogenic and non-pathogenic *E. coli* [75] but seems to play some role in ExPEC virulence. A chicken experimental model of systemic infection using mutant *E. coli* strains (defective for aerobactin and salmochelin) producing only enterobactin showed attenuation of virulence [86,87]. The inefficacy has been attributed to the absorption of this protein by host immune defense proteins, such as human neutrophil gelatinase-associated lipocalin (NGAL). It has been demonstrated that modification of enterobactin through glycosylation by IroB results in the generation of salmochelins that can evade sequestration by NGAL [88]. Enterobactin glycosylation is, therefore, an important virulence mechanism through which certain pathogenic strains can evade host immune defenses and obtain iron [89].

#### 5.3.2. Salmochelin

Salmochelin is found in ExPEC strains and is considered a characteristic virulence factor for this pathotype. It is encoded by the *iroBCDEN* gene cluster [90] located on *ColV* or *ColBM* virulence plasmids or identified on pathogenicity-associated islands (PAI) [91]. It has been reported that *iroB* is the sole gene with glycosyltransferase activity necessary for salmochelin production, which leads to glycosylation of enterobactin that changes its properties from strongly hydrophobic to hydrophilic. This chemical rearrangement may contribute to the virulence of ExPEC [92,93].

The IroB glycosyltransferase transfers glucosyl groups from uridine-50-diphosphoglucose to C5 residues of enterobactin 2,3-DHB [94], generating three types of cyclic salmochelins: monoglucosyl-C-enterobactin (MGE), diglucosyl-C-enterobactin (DGE), and triglucosyl-C-enterobactin (TGE). Only IroB is required for the conversion of enterobactin into salmochelins [90,94]. However, six different linear forms of salmochelins can also be generated from cyclic salmochelins by the IroD, IroE, and Fes esterases [94,95]. The IroN salmochelin receptor plays a role in virulence of UTI strain CP9 and NMEC strain S88 [96,97,98,99] and the *iroN* gene is overexpressed in intracellular bacterial communities (IBC) found in urothelial cells infected with UPEC strain UTI89 in a mouse model of UTI [100]. Furthermore, deletion of the *IroA* locus in APEC strain x7122 resulted in decreased virulence in chicken experimental models [87].

#### 5.3.3. Aerobactin

Aerobactin is another siderophore commonly harbored in ExPECs. Similar to salmochelin, this siderophore is also encoded by *ColV* and *ColBM* plasmids [27,91] and its biosynthesis is driven by enzymes encoded by the *iucABCD* (iron uptake chelate) genes [101]. From L-lysine, IucD monooxygenase forms N6-hydroxy-L-lysine [102]. Then, IucB acetyltransferase synthesizes N6-acetyl-N6-hydroxy-Llysine from N6-hydroxy-L-lysine and acetyl-CoA [103]. IucA and IucC, respectively, participate in the acylation and condensation of N6-acetyl-N6-hydroxy-Llysine to produce aerobactin [104,105,106].

Compared to commensal strains, aerobactin biosynthetic genes are more frequently detected in pathogenic *E. coli* strains isolated from food-producing animals [107,108,109]. Moreover, compared to the wild-type, the virulence of an APEC strain, deficient in aerobactin synthesis and uptake, is reduced in a chicken systemic infection model [87]. It has also been shown that aerobactin contributes to iron uptake *in vivo* in a UTI model [110]. Compared to enterobactin, very low aerobactin concentrations are sufficient to stimulate bacterial growth. Moreover, enterobactin binds to albumin in serum with a consequent low amount of iron sequestered from transferrin; iron uptake by aerobactin is more efficient since it can acquire more iron from transferrin than enterobactin [111,112,113].

#### 5.3.4. Yersiniabactin

ExPECs may also produce yersiniabactin, which was initially detected in *Yersinia pestis* (from which its name), mainly during colonization of the urinary tract. Yersiniabactin contributes to the pathogenicity of uropathogenic *E. coli* (UPEC), especially during colonization of the urinary tract, and may protect bacterial cells against host immune response [27,114,115]. It is synthesized by a mixed NRPS/PKS (polyketide synthase) system [116]. All the genes responsible for its biosynthesis are grouped in a gene cluster (MiBIG accession number: BGC0001055), except for the phosphopantetheylyl transferase that allows cysteines, salicylate, and malonyl precursors to bind to the assembly complex [115,116]. Briefly, salicylate is formed from chorismate by the Irp9 (YbtS) salicylate synthetase, it is then adenylated by YbtE to be transmitted to the NRPS/PKS system. The NRPS encoded by irp2 forms two thiazoline rings from cysteines. On the PKS encoded by *irp1*, a malonyl linker is incorporated and YbtU reduces one thiazoline ring to thiazolidine before cyclization and condensation of the final thiazoline ring. Then, the newly synthesized siderophore is released from the PKS thioesterase domain while the YbtT thioesterase performs its editing function by eliminating aberrant molecules [116].

Yersiniabactin is involved in establishing UTI, as it plays a significant role in iron uptake in vivo [110] and promotes biofilm formation in human urine at bladder level [117]. In addition, it also reduces ROS formation from polymorphonuclear leukocytes, monocytes, and macrophages by scavenging iron and preventing Haber–Weiss reactions [118]. Thus, yersiniabactin could play an important role in virulence by activating the host response and innate immune system [115].

### 5.4. Protectines/Serum Resistance

Protectines are a category of ExPEC’s virulence factors responsible for the characteristic serum resistance of these bacteria. Among all the known protectines, in this review, we focused on three resistance determinants: TraT, OmpA, and the capsular antigen K.

#### 5.4.1. TraT

TraT is one of the genes in the *tra* operon, a genetic unit involved in the transfer of the R factor, which is the conjugative plasmid (F plasmid) of *E. coli* [119,120]. The *traT* gene product is an outer membrane protein of 23 kDa (present at 20,000 to 30,000 copies per cell) noncovalently associated with peptidoglycan that behaves similarly to several peptidoglycan associated lipoproteins. It exists in the membrane as multimeric aggregates with a substantial portion exposed on the external face of the cell’s outer membrane [120,121,122,123,124]. TraT grants bacteria protection from the lytic action of complement and has several properties in common with other major outer membrane proteins, particularly the porins [123]. The oligomeric form of this protein is highly resistant to denaturation at temperatures below 70 to 80 °C, digestion by trypsin or pronase in outer membrane fractions, and solubilization by a variety of detergents [121,125].

The role of TraT in serum resistance is to inhibit the correct assembly or membrane insertion of the membrane attack complex of complement [126]. The TraT protein has also been reported to reduce the susceptibility of wild-type *E. coli* cells to phagocytosis by mouse peritoneal macrophages [127]. Another peculiar characteristic of the F plasmid in *E. coli* cells is the surface exclusion, defined as the reduced ability of a strain carrying a conjugative plasmid to act as a recipient in conjugation with cells carrying identical or closely related plasmids. Therefore, cells carrying the F plasmid typically have a 100- to 300-fold reduction in their ability to act as recipients relative to an F negative cell [120,128]. TraT protein blocks conjugation at an earlier stage before the cells have formed stable mating aggregates [121].

#### 5.4.2. OmpA

Outer membrane proteins (OMPs) and lipoproteins have many functions, including membrane structure and stability, signal transduction, active and passive ion, and solute transport, defense, and catalysis [129]. In addition, most OMPs are surface exposed and are potentially critical in bacteria interactions with the mammalian host and its defense mechanisms, bacteriophages, and other bacteria or microorganisms [130].

Outer membrane protein A (OmpA) is a major heat modifiable OMP in *E. coli* and is one of the most characterized OMPs. Its molecular mass is 33 kDa [131] and its structure is characterized by an N-terminal domain that forms an eight-stranded, anti-parallel β-barrel, which is embedded in the outer membrane noncovalently anchored to peptidoglycan and confers porin activity [132,133,134]. The C-terminal domain of OmpA is globular and located in the periplasmic space to interact with peptidoglycan [135,136]. OmpA has both structural and ion-permeable porin roles, with its ionic pore controlled by a salt-influenced electrostatic gating mechanism that allows bacterial survival during osmotic stress [137]. OmpA expression is closely associated with the expression of type 1 fimbriae, confirmed that *ompA* deleted mutants showed the suppression of type 1 fimbriae expression [138].

The importance of OmpA in pathogenicity has been established in numerous experimental models. Weiser and Gotschlich (1991) demonstrated that *ompA*-deficient *E. coli* K-1 (meningitic strain) was less virulent in a chick embryo and neonatal rat model and more sensitive to serum complement-mediated bactericidal effects [139]. OmpA is a virulence factor in meningitic strains of *E. coli* and has a function in adhesion to and invasion of the central nervous system, capillary endothelium, and astrocytes [140,141,142]. OmpA and its homologs are also involved in adhesion to epithelial cells on mucosal surfaces. Furthermore, OmpA mediates adhesion to leukocytes and macrophages in pathogenic strains of *E. coli* [130].

Through OmpA and its homologs, several pathogenic bacteria have mechanisms to escape host normal serum proteins and antibodies [143,144,145,146]. This is accomplished by binding the complement fluid-phase regulated protein C4B by *E. coli* (especially logarithmic-phase *E. coli*) [143]. OmpA-positive *E. coli* K1 induces the antiapoptotic factor Bclxl that blocks cytochrome C release and prevents macrophage apoptosis, thus assuring the survival of both macrophage and intracellular bacteria [147]. In addition, OmpA reduces proinflammatory cytokine production by interference with NF-kB activation [148,149].

Nicholson et al. (2009) demonstrated that OmpA is critical for promoting persistent infection within the epithelium [150]. Indeed, OmpA expression in wild-type bacteria was increased 20–30 fold between 16 and 24 h after infection during urinary tract infection. Furthermore, *E. coli ompA* deficient mutants adhered and invaded bladder epithelium significantly reduced bacterial load compared to wild-type strains [151]. *OmpA* deficient *E. coli* were more susceptible than the wild-type strain to neutrophil-derived antibacterial peptide lactoferricin (a lactoferrin-derived antimicrobial peptide) and pulmonary surfactant proteins A and D (collectins), which increase membrane permeability [152,153].

In NMEC the central role of OmpA in adherence and invasion was demonstrated using *ompA*-deficient mutants and concluding a 25–50 times reduction in invasion compared to non-deficient strains [140,141].

#### 5.4.3. Capsular Antigens

The bacterial capsule is an extracellular structure that microscopy can view as an extensive layer surrounding the cell [154]. The capsule is required to prevent the host immune system, and strains without capsules are usually non-pathogenic [155]. The presence of a capsule showed to be a key mechanism used by Gram-negative bacteria to evade phagocytosis [155].

The capsule is made up of long polysaccharide chains known as capsular polysaccharides (CPS), linear polymers of repeating carbohydrate subunits that sometimes also include a prominent amino acid or lipid component [156], which are typically negatively charged and generate a highly hydrated capsular layer. In some *E. coli* isolates, this layer can extend from the cell surface for approximately 100–400 nm [154]. Polyprenol-linked CPS repeat units are synthetized in the cytoplasm, flipped across the inner membrane by a Wzx protein, and polymerized into the full-length CPS by a Wzy protein. After export, the CPS chains are assembled into a capsule structure on the cell surface but it is not entirely clear how they are retained, and the process may be multifactorial [157].

The capsules of most ExPEC strains are thin, patchy, acidic, thermostable, and highly anionic, characteristics that identify group II polysaccharides [156,158]. Among over 80 different identified capsular (K) antigens [159] K1 and K2 are considered major determinants of ExPEC serum resistance [160,161]. The degree of serum resistance of K+ strains has been reported to be proportional to the amount of capsular material present [162] and to vary with the K type [163,164]. The role of K-capsule in serum resistance in vitro and in vivo within animal models is well-known [165,166,167]. The diversified structure of polysaccharide capsules produced by several dozen types of UPEC strains allows mimicking host tissue components and making immune system recognition difficult [35]. For example, *E. coli* strains expressing the capsular antigen are associated with the development of neonatal sepsis [168]. Capsular antigen K that covers the bacterial cell surface may inhibit adhesion to epithelial cells. The interaction of FimF with d-mannose inhibits the transcription of capsid genes, which may lead to a reduced amount of K antigen on the surface of the *E. coli* cell, and, as a result, facilitate the adhesion process [169].

While lipopolysaccharide (LPS) and other bacterial surface components readily bind C3b, and the complement proteins can penetrate a capsular layer, the presence of a CPS layer effectively masks the underlying opsonized cell surface [170]. However, even within a single capsular serotype (e.g., *E. coli* K1), the extent of protection from opsonization may vary [171]. In addition, capsular polysaccharide blocks opsonization as it interferes with complement deposition in a dose-dependent way [172,173].

Some CPSs do not elicit an immune response even when conjugated to a carrier protein [174], and for this reason, they are called molecular mimics because they have the same structure as human glycans [175]. For example, *E. coli* K1 CPSs consist of a 2,8-linked polysialic acid which is identical to the polysialic acid found on neuronal and immune cells [155]. Once bacteria are internalized, phagosome–lysosome fusion leads to lysis of the bacterial cell; however, some bacteria such as *E. coli* K1 can survive and multiply within phagocytic and epithelial cells [176,177,178,179]. Endosomes containing these encapsulated bacteria acquire endosomal markers, which are characteristic of the early and late phases of the endosome but do not fuse with lysosomes. The mechanism behind this is unknown but probably is due entirely to the presence of CPS as isogenic acapsular mutants are effectively degraded in lysosomes [155]. The degree of impairment of phagocytosis is proportional to the amount of polysaccharides [172]. Phagocytosis of K1 strains increases after exposure to anti-K1 antibodies [172,180] or disruption of capsular polysaccharides by heating [181]. The anti-complement activity of capsular polysaccharides may also increase the survival in serum of some encapsulated strains [182,183]. 

### 5.5. Toxins

The most frequently detected genes encoding toxins in ExPECs are: *hlyA*, *hlyD*, *hlyF* (α-hemolysin), *cnf1* (cytotoxic necrotizing factor 1), *sat* (secreted autotransporter toxin), *pic* (protease involved in colonization), and *vat* (vacuolating autotransporter protein) [184,185,186,187,188,189,190].

#### 5.5.1. α-Hemolysin

The hemolysin is a toxin expressed by the operon *hlyCABD*. The secreted HlyA protein is 110 kDa [191]. HlyA is post-transcriptionally heterogeneously modified through the enzymatic activity of hlyC. The acylation is required for hemolytic and cytotoxic activities, but the modification is not required for extracellular secretion. The immature (unacylated) and mature forms of the hemolysin are respectively referred to as proHlyA and HlyA. The *hlyB* and *hlyD* genes encode proteins involved in the cytoplasmically modified HlyA polypeptide secretion across the inner and outer membranes without amino-terminal cleavage of the polypeptide [191]. These proteins form a type I secretion complex together with the TolC unlinked gene product [192]. The secretion pathway of *E. coli* hemolysin is generally considered the prototypical type I secretion protein among Gram-negative bacteria [193].

*E. coli* hemolysin appears to be cytotoxic to many different host-cell types and towards many hosts, both animals and humans. The target and host-cell specificity could be due to the sequence and N-linked oligosaccharide-modification differences in the primary receptors for the toxins, β2 integrins, on immune cells [194,195,196]. The mechanism for *E. coli* hemolysin cytotoxicity involves the formation of membrane pores. Therefore, when erythrocytes are used as target cells, loss of intracellular K^+^ ions and subsequent influx of cations and water leads to osmotic lysis [197,198]. The initial pore formation leads to partial lysis of erythrocytes and subsequently triggers purinergic-receptor activation and creates a host-mediated pannexin pore. These events lead to the complete lysis of the cells.

There are several studies on the effects of the *E. coli* hemolysin on host cells *in vitro*, and it is difficult to assess which are relevant to the pathogenesis of UPEC. It seems unlikely that the lysis of erythrocytes is significant, although the canonical hypothesis is that the release of iron-containing heme molecules plays an important role in disease pathogenesis. As for the *E. coli* hemolysin activity against other host-cell types, several variables confound interpretations and conclusions about its role in uropathogenesis. The most significant variable is the presence of LPS in *E. coli* hemolysin preparations for *in vitro* experiments. In general, at low, sub-lytic, or apoptotic-inducing concentrations, the hemolysin triggers proinflammatory events [199,200,201].

#### 5.5.2. Cytotoxic Necrotizing Factor 1

CNF1 was initially described as a toxin causing dermonecrosis when CNF-producing *E. coli* strains were intradermally injected in rabbits. Historically, its first described cytotoxic activity was the formation of multi-nucleated cells in culture [202]. CNF1 is a 115 kDa protein that catalyzes the deamidation of a conserved-glutamine residue in three members of the Rho family of GTP-binding proteins. This leads to the activation of three specific GTPases: RhoA, Cdc42, and Rac [203,204]. The CNF1 protein is divided into three functional domains [205]. The enzymatic C-terminal half of the CNF1 polypeptide is present in the host cytosol after cleavage and release from late endosomes [206]. The very N-terminal CNF1 domain appears to be responsible for binding to a laminin receptor-precursor protein [207,208]. An internal CNF1 domain is responsible for translocation across the endosomal membrane [209]. The activation of these regulators leads to cytoskeleton rearrangements, cell cycle disruption, and interruption of host-cell signaling pathways [210].

Epidemiologically, CNF1 production is more often associated with UPEC strains responsible for more severe UTIs; Andreu et al. showed that 48% of the pyelonephritis isolates were *cnf1* positive [211]. As is the case for hemolysin, the role of CNF1 in uropathogenesis is not entirely known, despite the evidence that it induces neutrophil dysfunction and increased epithelial-cell invasion [212].

#### 5.5.3. Type V Secretion Family

The UPEC-toxin SPATE-family member that has received the greatest attention is the secreted autotransporter toxin (Sat). The *sat* gene is present, along with the hemolysin and pap pili determinants, within the large *pheV*–associated pathogenicity island of UPEC-model strain CFT073. The *sat* gene encodes a 142 kDa protein that possesses the three characteristic domains of SPATE proteins [213]. Guyer et al. demonstrated that Sat showed strong cytopathic effects on cultured cells and, in the murine model of UTI, histopathological lesions in the kidney can be ascribed to this protein [214]. In 2006 Maroncle et. Al. showed that a CFT073 *Sat* mutant did not have a statistically significant reduction in bacterial load either in the bladder or in the kidney compared to the parent strain CFT073and also showed, through the construction of active mutants, that Sat proteolytic activity was responsible for the cytotoxic activity, cytoskeleton rearrangements, and proteolysis of host proteins [213]. Moal et al. [215] showed that, in cultured HeLa cells, the Sat proteolytic activity initiates the disorganization of F-actin, resulting in detachment of the cell monolayers and loosening of cell-to-cell junctions. Furthermore, these events induce host-cell autophagy. Sat may also contribute to the exfoliation of the urothelial cells, an event commonly seen in UTIs model [212].

Two other SPATE family members, Pic and Vat, are commonly found in UPEC, and they were described in *Shigella flexneri* and APEC, respectively [216,217]. In UPEC model strain CFT073, the genes for these two autotransporter toxins (AT) are located respectively at the *aspV*- and *thrW*-associated pathogenicity islands. Pic possesses mucinase activity towards O-linked glycoproteins, such as CD43, CD45, and fractalkine, commonly found on neutrophil surfaces [218]. Vat is a vacuolating AT toxin that occurs in more than half of *E. coli* isolates causing cystitis and pyelonephritis [219]. There are no reports about the significance of Vat in UPEC in vitro or in vivo model systems. However, it has been demonstrated to be a significant virulence factor in APEC using respiratory- and cellulitis infection models in broiler chickens [217].

## 6. Antimicrobial Resistance (AMR)

AMR results in reduced efficacy of antimicrobials, making the treatment of patients costly and difficult, or even impossible [220]. In some cases, resistance extends to the therapeutic agents’ entire repository, posing a formidable challenge to antimicrobial therapy, bringing us back to the pre-antibiotic era [221,222].

Due to its particular ecology, *E. coli* can be considered a sensor of antimicrobial resistance. This organism has consolidated resistance traits that appeared many years ago, like TEM-1 β-lactamase, as well as the “newer β-lactamases” (Table 2) that consist of plasmid-mediated AmpC β-lactamases (e.g., CMY), extended-spectrum β-lactamases (e.g., CTX-M), and carbapenemases (e.g., NDM) [5,223,224,225].

In a 2014 report from the WHO, *E. coli* has been included in a list of the top nine microorganisms of international concern causing the most common infections in different settings: in the community, in hospitals, or transmitted through the food chain [222].

The cephalosporins, fluoroquinolones, and trimethoprim-sulfamethoxazole are often used to treat community and hospital *E. coli* infections, and resistance to these agents is responsible for delays in appropriate therapy with subsequently increasing morbidity and mortality [226,227]. Several surveillance studies during the 2000s across Europe, North and South America have shown that between 20 and 45% of ExPECs are resistant to first-line antibiotics, including cephalosporins, fluoroquinolones, and trimethoprim-sulfamethoxazole [228]. This problem undoubtedly must be controlled by a One Health approach, as the role of food-producing animals and the environment is not negligible [4,229]. β-Lactam antibiotics, especially the 3rd generation cephalosporins, are a major drug class used to treat severe community-onset or hospital-acquired infections involving *E. coli* [230]. Unfortunately, among *E. coli*, β-lactamase production remains the most important mediator of β-lactam resistance. β-lactamases are bacterial enzymes that inactivate β-lactam antibiotics by hydrolysis, resulting in the compound’s inefficiency [230]. These enzymes can hydrolyze penicillins, cephalosporins, and monobactams, but not the cephamycins and carbapenems, and are inhibited by traditional β-lactamase inhibitors clavulanic acid, sulbactam, and tazobactam [231]. 

Since the mid-2000s, CTX- M β-lactamases had been identified in different family members of *Enterobacteriaceae*, especially in *E. coli*, and have become the most widespread type of extended-spectrum β-lactamases (ESBL) [232]. Risk factors associated with infections caused by CTX-M-producing *E. coli* include the following: repeated UTIs, underlying renal pathology, previous use of antibiotics including cephalosporins and fluoroquinolones, previous hospitalization, diabetes mellitus, underlying liver pathology, and international travel to high-risk areas such as the Indian subcontinent [233].

*E. coli* possesses a chromosomal gene that encodes for an AmpC β-lactamase. Usually, low amounts of these β-lactamases are produced because the AmpC gene is regulated by a weak promoter and a strong attenuator [234]. AmpC enzymes are not inhibited by traditional β-lactamase inhibitors such as clavulanic acid, sulbactam, and tazobactam, although boronic acids and cloxacillin have shown a good level of inhibition [235]. Risk factors associated with infection of AmpC β-lactamase-producing bacteria are not as well defined as those associated with ESBL-producing bacteria [236].

A new type of metallo β-lactamase (MBL), named New Delhi metallo-β-lactamase-1 (NDM-1), was described in *K. pneumoniae* and *E. coli* recovered from a Swedish patient who was hospitalized in New Delhi, India [237]. MBLs can hydrolyze a wide variety of β-lactams, including penicillins, cephalosporins, and carbapenems, but not the monobactams (e.g., aztreonam), and are inhibited by metal chelators such as EDTA. Unfortunately, most NDM-1-producing bacteria are broadly resistant to various drug classes and carry various other resistance mechanisms (e.g., aminoglycosides and fluoroquinolones), leaving limited treatment options [221].

*Enterobacteriaceae* with NDM-1 have been recovered from many clinical settings, reflecting the disease spectra of these opportunistic bacteria, including hospital and community-onset UTIs, septicemia, pulmonary infections, peritonitis, device-associated infections, and soft tissue infections [221,238,239]. There is no evidence that NDM-1 producing *E. coli* are more virulent than other isolates; however, recent studies described the presence of NDM-1 β-lactamases in *E. coli* ST131 with an identical virulence genotype compared to ST131 producing CTX-M β-lactamases [240]. ST131 with VIM and KPC carbapenemases have also been described [241,242]. Due to the very resistant nature of these NDM-producing *E. coli*, the treatment of infections due to these bacteria will remain a great challenge. Antibiotics such as colistin, tigecycline, and fosfomycin show the best activity against NDM-producing bacteria [239]. However, Stone et al. (2011) described breakthrough bacteremia caused by *E. coli* that produce NDM while the patient was treated with tigecycline; the isolate developed resistance to tigecycline [243]. AMR is a major threat to public health, and its mechanisms of spread both in animals and humans still need to be addressed.

## 7. Conclusions

This review describes the wide spectrum of virulence factors deployed by ExPEC and emphasizes the potential pathogenic role of these pathogens both for humans and animals and the risk associated with the spread of AMR genes, harbored and transferred by these pathogens bacteria. The information may be helpful to characterize ExPEC isolates better and may be considered a starting point for future studies aiming to increase our knowledge on the relationship between ExPEC virulence factors and AMR applying a One Health approach.

## Figures and Tables

**Table 1:** Virulence genes, functions, and encoded virulence factors of ExPEC pathotype [27].

Description	Virulence Genes	Function	ExPEC Pathotype
Adhesins			
Type 1 fimbriae	*fim*	Factor of colonization in extraintestinal infections, biofilm formation	UPEC, NMEC, SEPEC, APEC
Afimbrial adhesin	*afa*	The non-fibrous adhesin binds to the DAF receptor on the cell surface epithelium as well as type IV collagen and members of the CEACAM family, hemagglutination capacity	UPEC
Dr fimbriae	*dra*	Binding to the DAF receptor on the surface epithelial cells as well as type IV collagen and members of the CEACAM family, mediation of internalization bacteria to the host cells	UPEC
P fimbriae	*pap*	Stimulate the production of cytokines by T lymphocytes, colonization factor in extraintestinal infections	UPEC, SEPEC, APEC
S fimbriae	*sfa*	Adhesion to intestinal epithelial cells, kidney, lower urinary tract cells; facilitate the penetration of bacteria into the tissues	UPEC, NMEC
F1C fimbriae	*foc*	Adhesion to renal epithelial cells and endothelial cells of the bladder and kidneys	UPEC
Iha	*iha*	Iron-regulated-gene-homolog adhesion	UPEC
Mat	*mat*	Meningitis associated and temperature regulated fimbriae	NMEC
Curli fiber gene	*crl, csg*	Enable biofilm formation and promote pathogenicity	UPEC, SEPEC, APEC
Antigen43	*agn43(flu)*	Protein of autotransporter family, adhesion and biofilm development	UPEC
Invasins			
Ibe ABC	*ibeA,B,C*	Cell invasion into the host tissues	NMEC, SEPEC, APEC
Iron uptake			
Aerobactin	*iuc, aer*	Siderophore, acquisition of Fe2^+^ / 3^+^ in the host system	UPEC, APEC
Iron repressible protein	*irp*	Yersiniabactin synthesis	NMEC
Salmochelin	*iroN*	Siderophore receptor, use of Fe ions obtained from the body host	UPEC, NMEC, SEPEC APEC
ChuA, Hma	*chu, hma*	Enable using of Fe from hemoglobin in the host system	UPEC, SEPEC
SitABC	*sitA,B,C*	Transportation of Fe, Mn	UPEC, APEC
Protectins/serum resistance			
Transfer protein	*traT*	Inhibition of the classical pathway of complement activity	NMEC, SEPEC APEC
Capsular antigens	*KpsMI-neuA, KpsMII*	The protection factor against phagocytosis and the spreading factor	NMEC, SEPEC
Outer membrane protein	*ompA*	Enable intracellular survival, evasion from the body’s defense.	UPEC, NMEC
Increased serum survival	*iss*	The protection factor against phagocytosis	NMEC, SEPEC, APEC
ColV, CvaC	*colV, cvaC*	Factor facilitating colonization	NMEC, SEPEC, APEC
Toxins			
Serin protease autotransporter	*pic*	Degrades mucins, facilitates colonization epithelium, damages of the cell membrane	UPEC
Secreted autotransporter toxin	*sat*	Proteolytic toxin, effect cytotoxic—influences on cell vacuolization	UPEC
Vacuolating autotransporter toxin	*vat*	Proteolytic toxin induces host cell vacuolization	UPEC, APEC
Hemolysin A	*hlyA*	Creation of pores in membranes of host cells (cell lysis)	UPEC
Cytotoxic necrotizing factor	*cnf*	Engaging in cell necrosis	UPEC, SEPEC
Cytolethal distending toxin	*cdt*	Cytolethal distending factor	SEPEC

**Table 2:** New β-lactamases found in *E. coli*. [15].

Enzymes	Classification	Examples	Spectrum of Resistance	Inhibition
Extended-spectrum β-lactamases(ESBLs)	Class A	CTX-M, TEM, SHV	Penicillins Cephalosporins Monobactams	Clavulanic acid, Avibactam, Vaborbactam, Relebactam, Tazobactam, Sulbactam
Plasmid-mediated AmpC β-lactamases	Class C	CMY, FOX, ACT, MOX ACC, DHA	Penicillins Cephalosporins Monobactams Cephamycins	Cloxacillin, Boronic acid
Metallo-β-lactamases(MBLs)	Class B	IMP, VIM, NDM	Penicillins Cephalosporins Cephamycins Carbapenems	Metal chelators, e.g., EDTA and dipicolinic acid
KPC carbapenemases	Class A	KPC	Penicillins Cephalosporins Cephamycins Carbapenems	Clavulanic acid (weak), Avibactam, Vaborbactam, Relebactam, Tazobactam, Boronic acid
OXA-β-lactamases	Class D	OXA-48, -181	Penicillins Temocillin β-lactamase combinations Carbapenems	Clavulanic acid, Avibactam, NaCl
